# High expression of heme oxygenase-1 in tumor-associated macrophages characterizes a poor-prognosis subtype in nasopharyngeal carcinoma

**DOI:** 10.18632/aging.202492

**Published:** 2021-02-11

**Authors:** Jianfeng Huang, Binbin Wan, Sha Li, Gang Liu, Qingfeng Pang, Jia Wu, Erwen Bao, Changling Sun, Yan Qin, Kewei Wang, Fei Yang, Yaxian Wu, Fuzheng Zhang, Bo Yang

**Affiliations:** 1 Department of Radiation Oncology, Affiliated Hospital of Jiangnan University, Wuxi 214062, Jiangsu Province, China; 2 Department of Physiopathology, Wuxi Medical School of Jiangnan University, Wuxi 214122, Jiangsu Province, China; 3 Department of Anesthesiology, Affiliated Hospital of Jiangnan University, Wuxi 214122, Jiangsu Province, China; 4 Department of Otolaryngology-Head and Neck Surgery, Affiliated Hospital of Jiangnan University, Wuxi 214062, Jiangsu Province, China; 5 Department of Pathology, Affiliated Hospital of Jiangnan University, Wuxi 214062, Jiangsu Province, China; 6 Department of Epidemiology, Affiliated Hospital of Jiangnan University, Wuxi 214062, Jiangsu Province, China; 7 Department of Radiation Oncology, University of Miami, Miami, FL 33136, USA

**Keywords:** heme oxygenase-1, CD163, tumor-associated macrophages, nasopharyngeal carcinoma, prognostic significance

## Abstract

Tumor-associated macrophages (TAMs) are important components of the tumor microenvironment, which are characterized by pro-tumor M2 phenotype and correlate with poor survival of nasopharyngeal carcinoma (NPC). Heme oxygenase-1 (HO-1) plays a crucial role in macrophage polarization toward M2 phenotype, but its prognosis significance in NPC has been rarely determined. To gain insights into the HO-1 expression profile and to determine the clinical significance of HO-1 in NPC, we performed immunohistochemistry analyses in 126 NPC specimens. CD163, a highly specific marker of M2 macrophages, was used as a surrogate for the polarization state of TAMs. Our results showed that high expression of HO-1 and CD163 were detected in TAMs for 57.9% (73/126) and 61.9% (78/126) of the studied patients, and both of them were significantly associated with worse survival. Additionally, a significant correlation between the intensities of HO-1 and CD163 was identified, and HO-1 exhibited a superior ability in predicting survival compared with CD163. Our study revealed for the first time that overexpression of HO-1 characterized a poor-prognosis subtype in NPC. Individualized therapy targeting HO-1 might serve as a promising treatment modality for NPC.

## INTRODUCTION

Nasopharyngeal carcinoma (NPC) is a highly invasive malignancy prevalent in Southern China, Southeast Asia, and North Africa. Worldwide, 129,079 cases of NPC were reported in 2018 [1]. The standard of care for non-metastatic NPC is radiotherapy for early-stage patients and chemoradiotherapy for locally advanced disease. However, 20–30% of the patients still develop local-regional recurrence and/or distant metastasis [2, 3]. Thus, newer treatment modalities, especially the use of molecular targeted therapeutics, are intensively sought.

Studies have shown that tumor microenvironment plays an important role in carcinogenesis and progression of cancer [4]. Macrophages are essential components of tumor stroma cells, which are extremely plastic cells. They could be polarized into two functionally distinct forms, M1 and M2 phenotypes [5], of which M1 macrophages contribute to mediating pro-inflammatory immune response and protecting against tumorigenesis, while M2 macrophages function in promoting immune evasion of tumor cells and producing immune tolerance. Consequently, M2 macrophages are widely considered as pro-tumor macrophages.

Tumor-associated macrophages (TAMs) are the most abundant immune cell populations in the tumor microenvironment, which are characterized by M2 phenotype and are found to promote the growth and invasion of cancer cells [6]. CD163 is a highly specific marker of M2 macrophages, and it was reported that high level of CD163^+^ TAMs was associated with poor survival in NPC [7]. Consequently, inhibiting macrophage polarization toward M2 phenotype is being closely scrutinized for a potential strategy in the treatment of NPC.

Heme oxygenase-1 (HO-1) is well-known as a stress-inducible protein in response to oxidative stress and inflammatory stimuli, owing to the anti-inflammatory and immunomodulatory properties [8]. Recently, researchers have reported that HO-1 plays a crucial role in polarization of M2 macrophages [9]. Moreover, HO-1 was found to be over-expressed in various tumors, and it is considered to be a key pro-tumor molecule against host attack as well as chemotherapy and radiation therapy [10–13]. Shi et al. examined HO-1 expression using RT-PCR in 32 NPC patients, and the results showed that HO-1 expression was found in 19 patients (59.4%), among which nine (47.4%) showed no response to radiotherapy, while among the 13 patients with HO-1 negative expression 12 (92.3%) exhibited responsiveness to radiotherapy, suggesting that high expression of HO-1 might correlate with poor response to radiotherapy [14]. However, this study was done with rather small number of patients and short follow-up periods, thus largely restricting the interpretation of the prognosis significances, especially in survival and disease progression of HO-1 in NPC.

In the current study, 126 newly diagnosed, non-metastatic NPC patients with consecutive follow-ups were reviewed retrospectively with the primary goal of determining the long-term prognostic importance of HO-1 expression in NPC. In addition, the interrelationship between HO-1 expression and CD163 expression in NPC was explored as a secondary goal to examine if there exists a role for HO-1 in TAMs polarization.

## RESULTS

### Patient characteristics

Of the 126 patients included in this study, 100 (79.4%) were male and 26 (20.6%) were female; the median age was 51 years (range, 22-80 years).There were 5 (4.0%) patients presented with stage I disease, 22(17.4%) patients with stage II disease, 66 (52.4%) patients with stage III disease, and 33 (26.2%) patients with stage IVA disease. The studied cohort consisted of patients of same ethnicity (Han Chinese) and provincial affiliation (Jiangsu Province, China). All patients underwent definitive IMRT with a total dose of 66-76 Gy to the primary tumor and involved cervical lymph nodes, 60 Gy to the high-risk areas, and 50-54 Gy to the low-risk volumes. The treatment was delivered with conventional fractionation scheme: once daily and five fractions per week. Platinum-based induction or/and concurrent chemotherapy was administered for 114 (90.5%) patients: 54 with induction chemotherapy, 35 with induction plus concurrent chemotherapy, and 25 with concurrent chemotherapy alone. Detailed patient information is shown in Table 1.

**Table 1:** Patient characteristics of the studied cohort (n=126).

**Variables**	**n (%)**
Sex, male/female	100 (79.4)/26 (20.6)
Age, years [median (range)]	51 (22-80)
T classification, T1/T2/T3/T4	24 (19.0)/49 (38.9)/35 (27.8)/18 (14.3)
N classification, N0/N1/N2/N3	12 (9.5)/22 (17.5)/70(55.6)/22 (17.5)
Clinical stage, I/II/III/IVA	5 (4.0)/22 (17.5)/66 (52.4)/33(26.2)
WHO histological type, II/III	96(76.2)/30(23.8)
Chemotherapy modalities, IC/IC+CCT/CCT	54 (42.9)/35 (27.8)/25 (19.8)
Follow-up duration, months [median (range)]	49.0 (8.9-111.4)

Abbreviations: n, number of patients; T, tumor; N, lymph node; IC, induction chemotherapy; CCT, concurrent chemotherapy.

With a median follow-up of 49.0 months (range: 8.9-111.4 months), locoregional recurrence and distant metastasis were observed in 18 and 24 patients, respectively, and 26 of them deceased of disease progression, among which 11 presented with locoregional recurrence and 15 presented with distant metastasis. The 4-year LRFFS, DMFS, PFS and OS in our cohort were 91.3%, 79.2%, 74.3% and 81.3%, respectively.

### HO-1 and CD163 expression in NPC specimens

**Figure 1:**
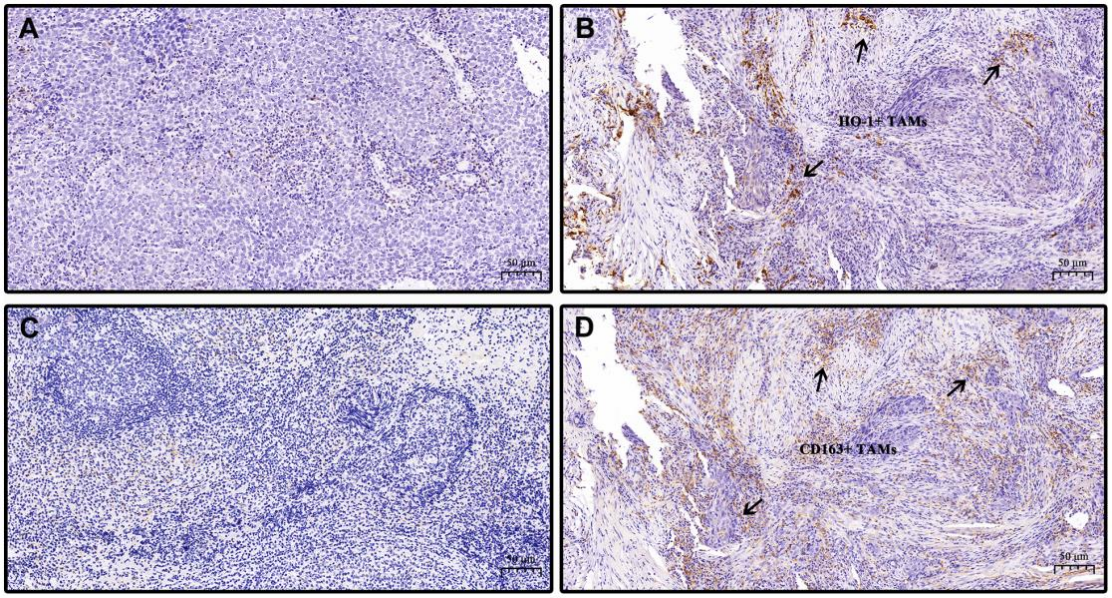
**Representative specimens showing low and high expression of HO-1 and CD163 in nasopharyngeal carcinoma tissues.** Immunohistochemical staining of HO-1 and CD163 are presented. HO-1 and CD163 immunohistochemical scoring: score 0 (no staining), score 1 (weak staining), score 2 (moderate staining), score 3 (strong staining). Staining intensities with scores no greater than 1 were defined as low expression while as high expression when scores are above 1. (**A**) low expression of HO-1. (**B**) high expression of HO-1. (**C**) low expression of CD163. (**D**) high expression of CD163. (**B**, **D**) are from the same section view of tumor specimen of the same patient. Arrows point out co-expression of HO-1 and CD163 in tumor-associated macrophages (TAMs). Scale bar, 50 μm. Magnification, 20×.

**Figure 2:**
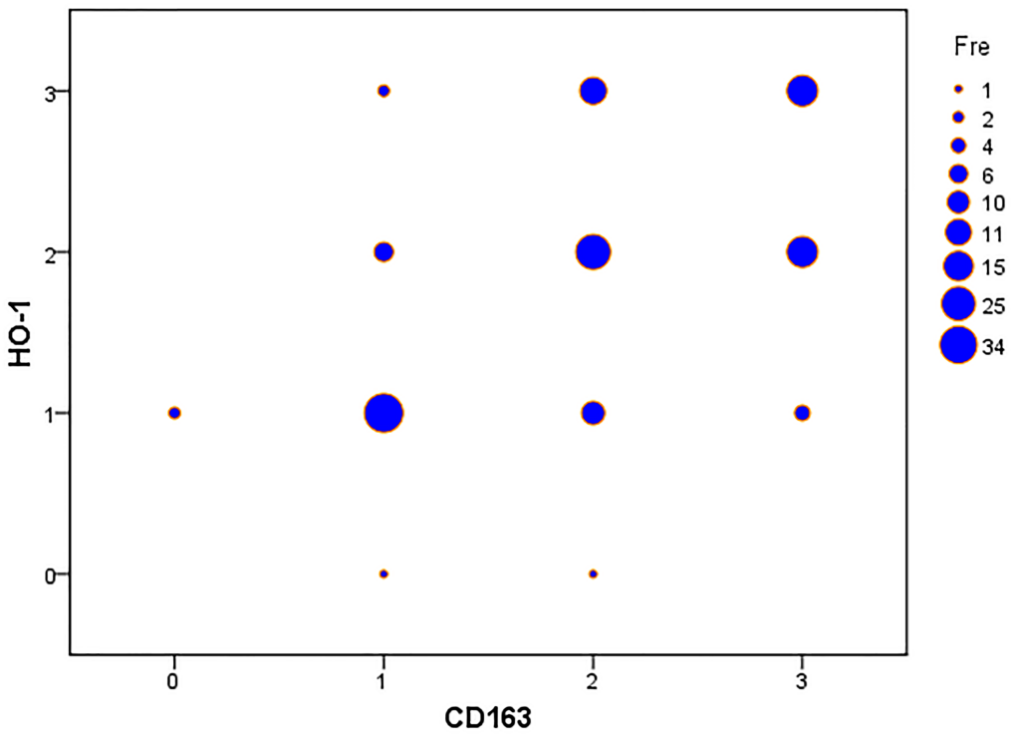
**HO-1 immunohistochemical scoring significantly correlates with expression of CD163 in nasopharyngeal carcinoma tissues.** Spearman’s correlation analysis demonstrates a significant correlation between the expression levels of HO-1 and CD163. The Spearman’s correlation coefficient is 0.536, p-value < 0.001.

High expression of HO-1 and CD163 were detected in 57.9% (73/126) and 61.9% (78/126), respectively, of the studied cohort. Considerable amounts of TAMs exhibited co-expression of HO-1 and CD163 (Figure 1B, 1D). HO-1 was mainly found in the cytoplasm and nuclei of TAMs, while CD163 was observed in the TAMs by a granular cytoplasm or a cytoplasmic and membrane staining pattern. Table 2 summarizes the distribution of HO-1 and CD163 scoring within the clinicopathological characteristics of patients. The initial cohort was divided into two groups defined a priori based on expression levels of HO-1 or CD163: patients with high expression (HO-1^high^; CD163^high^) and patients with low expression (HO-1^low^; CD163^low^). The results showed that the expression of CD163 was significantly higher in patients with undifferentiated non-keratinized carcinoma than those with differentiated carcinoma (*P* = 0.019). However, neither HO-1 nor CD163 correlated with gender, age, T stage, N stage and clinical stage of patients (*P*>0.05). Interestingly, our study also demonstrated that there was a moderately significant correlation between the expression levels of HO-1 and CD163 in TAMs with a Spearman’s correlation coefficient of 0.536 (*P* < 0.001) (Figure 2).

**Table 2:** Distribution of HO-1 and CD163 expression within the clinicopathological characteristics of nasopharyngeal carcinoma patients.

**Parameters**	**n**	**HO-1**			**CD163**		
		**Low (n=53)**	**High (n=73)**	**p-value**	**Low (n=48)**	**High (n=78)**	**p-value**
**Gender**				0.977			0.620
Male	100	42	58		37	63	
Female	26	11	15		11	15	
**Age (years)**				0.931			0.495
≤51	66	28	38		27	39	
>51	60	25	35		21	39	
**T stage**				0.636			0.944
T1–T2	73	32	41		28	45	
T3–T4	53	21	32		20	33	
**N stage**				0.970			0.854
N0,1,2	104	43	61		40	64	
N3	22	9	13		8	14	
**Clinical stage**				0.422			0.984
I–III	92	36	56		35	57	
IVA	34	16	18		13	21	
**Histological type**				0.125			0.019
II	96	44	52		42	54	
III	30	9	21		6	24	

Abbreviations: HO-1, Heme oxygenase-1; n, number of patients; T, tumor; N, lymph node.

### Prognostic factors of NPC patients

**Figure 3:**
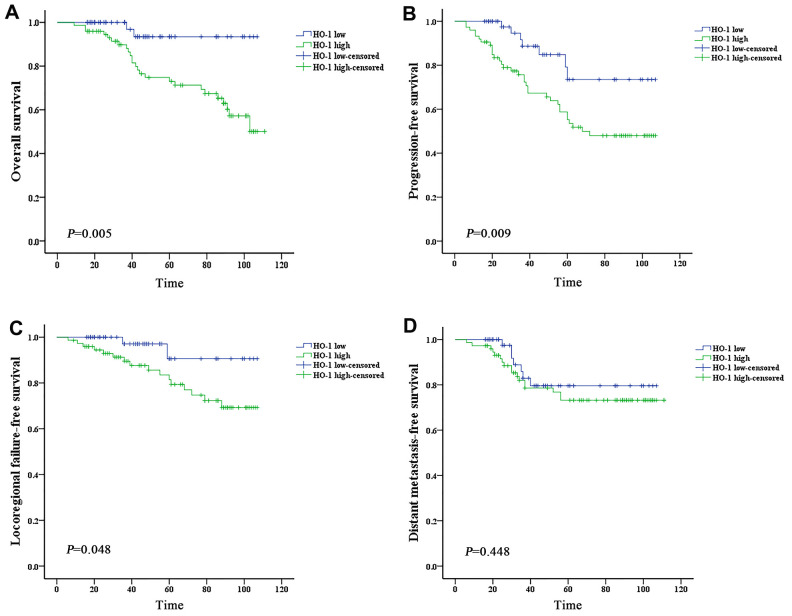
**High expression of HO-1 is associated with worse prognosis of nasopharyngeal carcinoma.** Survival was estimated by the Kaplan-Meier method and compared with log-rank tests. (**A**) The HO-1 high group exhibited significantly lower overall survival than the HO-1 low group (log-rank p-value = 0.005). (**B**) The HO-1 high group exhibited significantly lower progression-free survival than the HO-1 low group (log-rank p-value = 0.009). (**C**) The HO-1 high group exhibited significantly lower local-regional failure-free survival than the HO-1 low group (log-rank p-value = 0.048). (**D**) The HO-1 high group exhibited lower distant metastasis-free survival than the HO-1 low group, but the difference is not statistically significant (log-rank p-value = 0.448).

**Figure 4:**
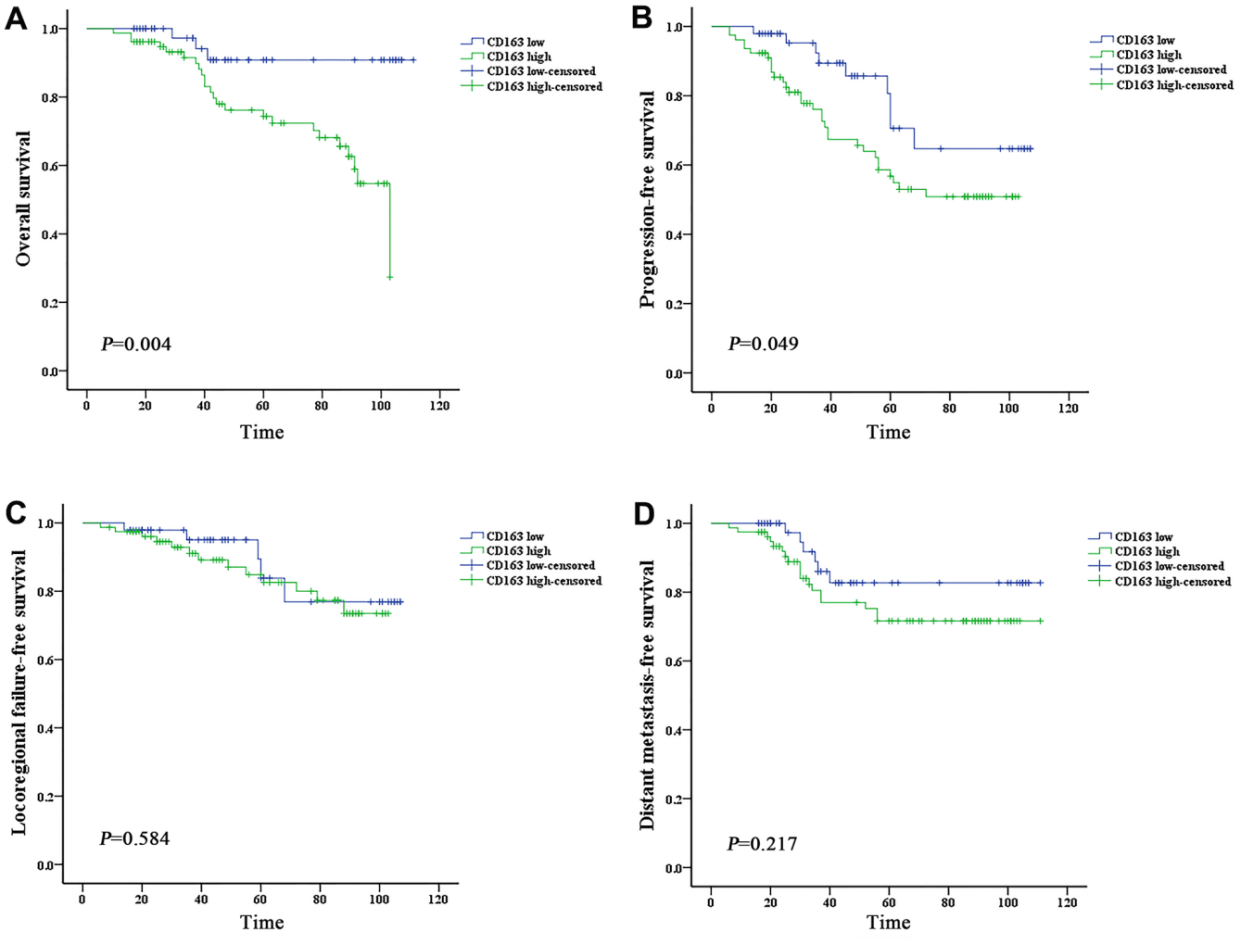
**High expression of CD163 is associated with worse prognosis of nasopharyngeal carcinoma.** Survival was estimated by the Kaplan-Meier method and compared with log-rank tests. (**A**) The CD163 high group exhibited significantly lower overall survival than the CD163 low group (log-rank p-value = 0.004). (**B**) The CD163 high group exhibited significantly lower progression-free survival than the CD163 low group (log-rank p-value = 0.049). (**C**, **D**) The CD163 high group exhibited lower local-regional failure-free survival and distant metastasis-free survival than the CD163 low group, but the differences are not statistically significant (log-rank p-values>0.05).

Subgroups analysis was performed to discriminate the potential prognostic factors on predicting survival. Both univariate and multivariate analysis revealed that patients with high expression of HO-1 or CD163 showed worse OS and PFS than those with low scoring (*P* < 0.05) (Tables 3, 4 and Figures 3A, 3B, 4A, 4B). The 4-year OS and PFS of the HO-1^high^ group (74.8% and 67.3%, respectively) were significantly lower than those of the HO-1^low^ group (93.4% and 84.8%, respectively) with *P* values less than 0.05. The 4-year OS and PFS between CD163^high^ and CD163^low^ groups were 76.2% versus 90.8% (*P* = 0.006) and 67.4% versus 85.7% (*P* = 0.033), respectively. Results of survival analysis also indicated that higher HO-1 expression was associated with lower LRFFS of patients (*P* = 0.048) (Figure 3C). However, no significant correlation was found between the expression levels of HO-1 or CD163 and DMFS (*P*>0.05) (Figure 3D, 4D). In addition, independent prognostic factors for PFS were also examined among other groups of patients with different N stage (N_0-2_ vs N_3_, *P* = 0.005) and clinical stage (stage I–III vs stage IVA, *P* = 0.007), and stage IVA patients demonstrated a worse OS than those with stage I–III (*P* = 0.012).

**Table 3:** Impact of prognostic factors on OS of nasopharyngeal carcinoma by univariate and multivariate analysis.

**Covariate**	**Univariate**		**Multivariate**	
	**HR (95% CI)**	***p*-value**	**HR (95% CI)**	***p*-value**
**Gender**				
Male	Reference			
Female	0.99 (0.34-2.90)	0.978		
**Age (years)**				
≤51	Reference			
>51	1.34 (0.63-2.87)	0.453		
**T stage**				
T1–T2	Reference			
T3–T4	0.72 (0.34-1.55)	0.402		
**N stage**				
N0,1,2	Reference		Reference	
N3	4.48(1.05-19.13)	0.043	3.48(0.77-15.68)	0.104
**Clinical stage**				
I–III	Reference		Reference	
IVA	6.75 (1.52-29.90)	0.012	2.70(1.01-7.18)	0.047
**Concurrent chemotherapy**				
No	Reference			
Yes	2.04(0.78-5.35)	0.146		
**HO-1**				
Low	Reference		Reference	
High	5.24 (1.23-22.29)	0.025	3.01(1.23-7.39)	0.016
**CD163**				
Low	Reference		Reference	
High	5.64 (1.64-19.32)	0.006	3.91 (1.06-14.38)	0.040

Abbreviations: OS, overall survival; T, tumor; N, lymph node; HO-1, Heme oxygenase-1; HR, hazard ratio; CI, confidence interval.

**Table 4:** Impact of prognostic factors on PFS of nasopharyngeal carcinoma by univariate and multivariate analysis.

**Covariate**	**Univariate**		**Multivariate**	
	**HR (95% CI)**	***p*-value**	**HR (95% CI)**	***p*-value**
**Gender**				
Male	Reference			
Female	0.75 (0.33-1.71)	0.497		
**Age (years)**				
≤51	Reference			
>51	1.31 (0.70-2.44)	0.394		
**T stage**				
T1–T2	Reference			
T3–T4	0.55 (0.20-1.55)	0.260		
**N stage**				
N0,1,2	Reference		Reference	
N3	4.41(1.57-12.44)	0.005	3.78(1.31-10.86)	0.014
**Clinical stage**				
I–III	Reference		Reference	
IV	3.65 (1.43-9.35)	0.007	3.03(1.33-8.09)	0.027
**Concurrent chemotherapy**				
No	Reference			
Yes	1.50(0.72-3.13)	0.277		
**HO-1**				
Low	Reference		Reference	
High	3.27 (1.51-7.07)	0.003	3.34 (1.09-7.54)	0.010
**CD163**				
Low	Reference		Reference	
High	2.27(1.08-4.76)	0.030	2.12(1.01-4.45)	0.046

Abbreviations: PFS, progression-free survival; T, tumor; N, lymph node; HO-1, Heme oxygenase-1; HR, hazard ratio; CI, confidence interval.

### ROC analysis

**Figure 5:**
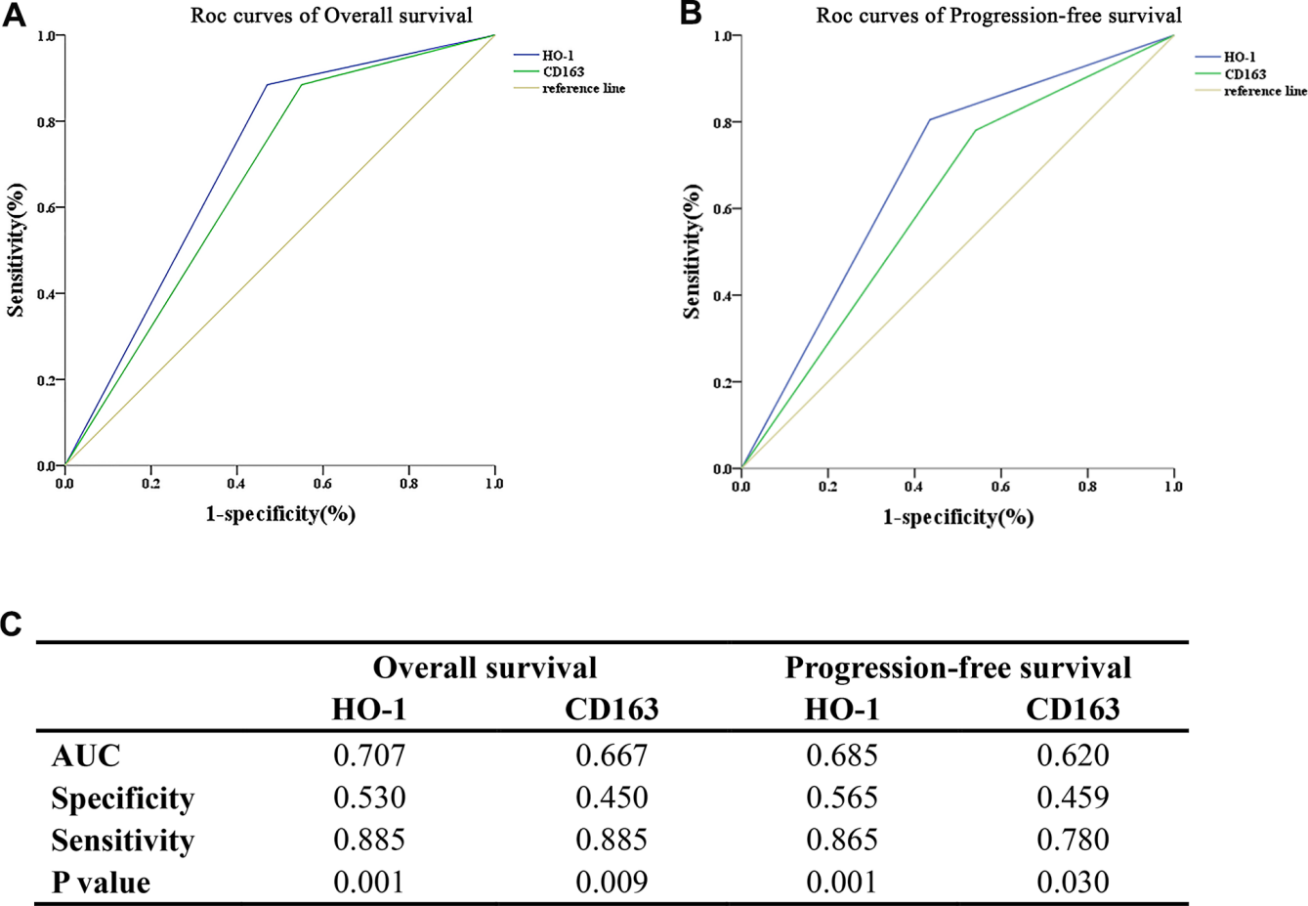
**HO-1 is a superior predictor for survival compared with CD163 in the studied cohort.** The predictive capability of HO-1 and CD163 for survival is accessed by the area under the curve (AUC) of the receiver operating characteristic (ROC) curves. (**A**) HO-1 exhibits a superior ability in predicting overall survival compared with CD163. (**B**) HO-1 exhibits a superior ability in predicting progression-free survival compared with CD163. (**C**) The AUC values of HO-1 in predicting OS and PFS of patients were 0.707 and 0.685, which are slightly higher than those of CD163 (0.667 and 0.620).

To further assess the predictive capability of HO-1 and CD163 for OS and PFS in NPC, the area under the curve (AUC) for the ROC curves were evaluated (Figure 5). These results showed that the AUC values of HO-1 in predicting OS and PFS of patients were 0.707 and 0.685, respectively, overperforming those of CD163 (0.667 and 0.620).

## DISCUSSION

In this retrospective study, we collected clinical information of 126 patients with non-metastatic NPC and examined the expression of HO-1 and CD163 proteins in pretreatment biopsy samples to determine their relationship with prognosis. The results showed that there was a significant correlation between the intensities of HO-1 and CD163, and both of them correlated with poor prognosis of NPC.

The prognostic significance of TAMs in cancer remains the subject of debate. High expression of TAMs was examined as a poor prognostic indicator in most studies [7, 15–18], however, other researchers found that TAMs could exert an antitumoral effect and confer a survival advantage [19–21]. The reasons for this discrepancy may be ascribed in part to the fact that different macrophage subsets were detected in these studies. In our study, CD163 protein, a highly specific marker of M2 macrophages, was used to examine the polarization state of TAMs. The results showed that high level of CD163 was significantly associated with worse OS and PFS in NPC patients, which was in agreement with a recent study conducted by Yu et al [7]. These findings together indicate that M2 TAMs may play a vital role in carcinogenesis and aggressiveness of NPC.

HO-1 can drive macrophage polarization toward M2 phenotype and promote tumor progression by regulating the tumor microenvironment [9, 10, 22–24]. In the present study, we reported in the first time that excessive expression of HO-1 in TAMs was associated with poor survival in NPC. The 4-year OS in HO-1^high^ and HO-1^low^ groups were 74.8% and 93.4% (*P* = 0.002), respectively. It is further noteworthy that, for the cohort studied, higher expression of HO-1 was associated with higher risk of local-regional recurrence (*P*=0.048), suggesting that a more intensive local treatment may need to be considered for the HO-1^high^ subtype.

**Figure 6:**
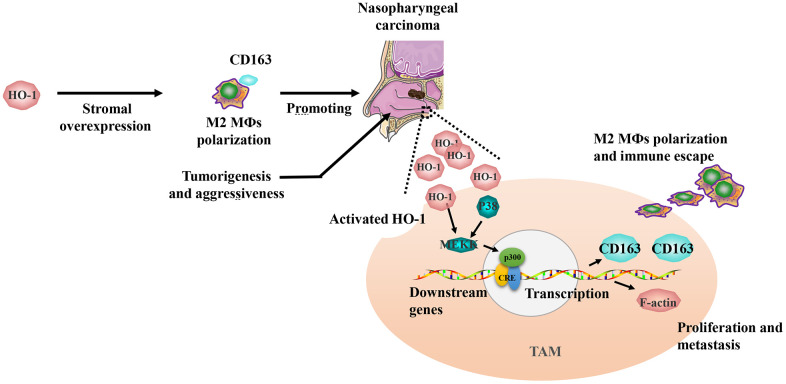
**Schematic diagram of HO-1 regulating CD163 expression and tumor-associated macrophage (TAM) polarization in the progression of nasopharyngeal carcinoma.** The overexpression of HO-1 could promote CD163 expression, and drive TAM polarization toward M2 phenotype. Finally, M2 TAMs related immune escape promotes the progression of nasopharyngeal carcinoma.

Interestingly, our study also found that considerable amounts of TAMs exhibited co-expression of HO-1 and CD163, and Spearman’s correlation analysis revealed a significant correlation between the expression levels of HO-1 and CD163. Combined with the phenomenon that M2 macrophage polarization could be induced by HO-1 overexpression [14], the correlation of HO-1 and CD163 might be involved in M2 TAMs activation and tumor aggressiveness in NPC (Figure 6). Additionally, the results of ROC analysis revealed that HO-1 was a superior predictor for OS and PFS as compared to CD163. Based on these findings, the strategy of targeting TAMs by inhibiting HO-1 activity appears to be promising for the patients with HO-1 overexpression.

Taken together, our study revealed that overexpression of HO-1 characterized a poor-prognosis NPC subtype. In addition, a significant correlation between the intensities of HO-1 and CD163 in TAMs was found, and HO-1 exhibited a superior ability in predicting OS and PFS compared with CD163. These findings suggested that individualized therapy targeting HO-1 might serve as a promising treatment modality for NPC.

## MATERIALS AND METHODS

### Patient population

Inclusion criteria were as follows: (1) newly diagnosed, non-metastatic NPC with histological confirmation of squamous cell carcinoma; (2) aged between 18–80 years old; (3) treated by definitive intensity-modulated radiotherapy (IMRT) with or without chemotherapy (≥2 cycles of platinum-based induction or/and concurrent chemotherapy) at initial diagnosis; (4) no concomitant autoimmune or inflammatory disease; (5) no secondary malignancy; (6) pretreatment Eastern Cooperative Oncology Group (ECOG) performance status 2 or lower.

A total of 126 eligible patients treated at the Affiliated Hospital of Jiangnan University between December 2008 and October 2018 were enrolled in the study. This study was reviewed and approved by the Ethics Committee of the Affiliated Hospital of Jiangnan University (LS2018071). Written informed consent for using clinical data and NPC tissues was obtained from all participants.

### Data collection

Medical records of the 126 patients were reviewed retrospectively. The following clinical data were abstracted: age, gender, histological type, stage, and treatment regimens. All patients were restaged according to the eighth edition of the American Joint Committee on Cancer (AJCC) staging system [25]. Collection of high-quality, paraffin-embedded NPC tissues from the original diagnostic biopsy was performed by a pathologist, and the survival data of patients were collected by a radiation oncologist.

### Immunohistochemical staining

Immunohistochemistry was performed to detect HO-1 and CD163 proteins in NPC tissues according to standard protocols. Briefly, 4 μm thick tissue sections were de-waxed and rehydrated, and heat-induced antigen recovery was performed in sodium citrate buffer (10 mM, pH = 6.0). All slides were incubated in 3% H_2_O_2_ to block endogenous peroxidase activity. The sections were then incubated with primary antibodies of HO-1 (1:500; Abcam, UK) and CD163 (1:500; Abcam, UK) at 4° C overnight. Phosphate buffer saline (PBS) was used as a negative control. After washing in PBS, the sections were incubated with biotinylated secondary antibodies (Kangwei, China) for 60 min at room temperature. Nuclei were stained with hematoxylin about 1 min. Antigenic sites were visualized using DAB kits (Kangwei, China).

### Evaluation of staining

Histochemical sections stained with immunohisto-chemistry were reviewed and scored by two experienced pathologists with discrepancies, if any, being resolved by consensus. The staining intensities of HO-1 and CD163 were scored on a 0-3 scale (0 = no staining, 1 = weak staining, 2 = moderate staining, 3 = strong staining). Similar to previous convention [7], staining intensities with scores no greater than 1 were defined as low expression while as high expression when scores are above 1 (Figure 1).

### Statistical analysis

Statistical analysis was performed using the IBM SPSS software, version 20.0 (IBM Corporation, Armonk, NY, USA). The chi-square test (χ^2^) was used to interrogate the association between dichotomous variables. Spearman’s correlation analysis was used to examine the correlation between the expression levels of HO-1 and CD163 proteins. Overall survival (OS), progression-free survival (PFS), distant metastasis-free survival (DMFS), and local-regional failure-free survival (LRFFS) were estimated by the Kaplan-Meier method and compared with log-rank tests. OS was calculated from the date of histological confirmation to the date of death or the last follow-up. PFS was defined as the time between diagnosis and the first occurrence of locoregional or distant recurrence or the last follow-up date. Multivariate analysis was performed using the Cox proportional hazard model. The receiver operating characteristic (ROC) curve was used to evaluate the predictive value of HO-1 and CD163 for the prognosis of NPC. The two-tailed p-values less than 0.05 are considered to be statistically significant.
